# The need for standardisation in life science research - an approach to excellence and trust.

**DOI:** 10.12688/f1000research.27500.2

**Published:** 2021-05-10

**Authors:** Susanne Hollmann, Andreas Kremer, Špela Baebler, Christophe Trefois, Kristina Gruden, Witold R. Rudnicki, Weida Tong, Aleksandra Gruca, Erik Bongcam-Rudloff, Chris T. Evelo, Alina Nechyporenko, Marcus Frohme, David Šafránek, Babette Regierer, Domenica D'Elia

**Affiliations:** 1 Faculty of Science, University of Potsdam, Potsdam, Brandenburg, 14476, Germany; 2 SB Science Management UG (Haftungsbeschränkt), Berlin, Berlin, 12163, Germany; 3 Information Technology for Translational Medicine S.A. ITTM S.A., Esch-sur-Alzette, Esch, 4354, Luxembourg; 4 Department of Biotechnology and Systems Biology, National Institute of Biology, Ljubljana, 1000, Slovenia; 5 Luxembourg Centre for Systems Biomedicine, University of Luxembourg, Esch-sur-Alzette, 4367, Luxembourg; 6 Institute of Computer Science, University of Białystok, Białystok, 15-328, Poland; 7 Division of Bioinformatics and Biostatistics, National Center for Toxicological Research, US Food and Drug Administration, Jefferson, AR, Jefferson, USA; 8 Department of Computer Networks and Systems, Silesian University of Technology, Gliwice, 44-100, Poland; 9 Department of Animal Breeding and Genetics, Bioinformatics section, University of Agricultural Sciences, Uppsala, 750 07, Sweden; 10 Department of Bioinformatics - BiGCaT, Maastricht University, Maastricht, 6229 ER, The Netherlands; 11 Maastricht Centre for Systems Biology (MaCSBio), Maastricht University, Maastricht, 6229 ER, The Netherlands; 12 Department of Systems Engineering, Kharkiv National University of Radio Electronics, Kharkiv Oblast, 61000, Ukraine; 13 Division Molecular Biotechnology and Functional Genomics, Technical University of Applied Sciences Wildau, Wildau, Brandenburg, 15745, Germany; 14 Masaryk University, Brno, 601 77, Czech Republic; 15 Leibniz Institute of Vegetable and Ornamental Crops (IGZ), Großbeeren, Brandenburg, 14979, Germany; 16 Institute for Biomedical Technologies, National Research Council, Italy, Bari, 70126, Italy

**Keywords:** Open Data, Open Access, Open Science, FAIR Principles, Standardisation, Education, Quality Management

## Abstract

Today, academic researchers benefit from the changes driven by digital technologies and the enormous growth of knowledge and data, on globalisation, enlargement of the scientific community, and the linkage between different scientific communities and the society. To fully benefit from this development, however, information needs to be shared openly and transparently. Digitalisation plays a major role here because it permeates all areas of business, science and society and is one of the key drivers for innovation and international cooperation. To address the resulting opportunities, the EU promotes the development and use of collaborative ways to produce and share knowledge and data as early as possible in the research process, but also to appropriately secure results with the European strategy for Open Science (OS). It is now widely recognised that making research results more accessible to all societal actors contributes to more effective and efficient science; it also serves as a boost for innovation in the public and private sectors. However  for research data to be findable, accessible, interoperable and reusable the use of standards is essential. At the metadata level, considerable efforts in standardisation have already been made (e.g. Data Management Plan and FAIR Principle etc.), whereas in context with the raw data these fundamental efforts are still fragmented and in some cases completely missing. The CHARME consortium, funded by the European Cooperation in Science and Technology (COST) Agency, has identified needs and gaps in the field of standardisation in the life sciences and also discussed potential hurdles for implementation of standards in current practice. Here, the authors suggest four measures in response to current challenges to ensure a high quality of life science research data and their re-usability for research and innovation.

## Introduction

Life science research is the driver for biotechnological applications, one of the fastest growing and potentially biggest future markets worldwide. As a result of advances in biological research and the support provided by information and communication technologies (ICT), new technologies have been developed that are changing our lives for example, by

- improving healthcare (e.g. new prognostic and diagnostic tools, improved disease prevention strategies and treatments, nutritional health, exercise and risk evaluation, toxicology);
- contributing to climate change mitigation and adaptation (e.g. by new fuels, new raw and renewable materials);
- increasing the efficiency and sustainability of farming and food production (e.g. by improved crops, artificial meat, life stock, new fertilising and watering strategies as well as plant protection solutions);
- using microorganisms as alternative production systems (e.g. using synthetic biology);
- decreasing waste production (e.g. by the production of biodegradable materials);
- modelling approaches leading to an increased understanding of how components in different systems interact and behave (e.g. by COMBINE, LiSyM);

These changes are directly connected to and driven by various factors such as globalisation, digitalisation and technology implementation. Digitalisation, in particular, plays a major role here because it permeates all areas of business, science, and society; and it is one of the key drivers for innovation and international cooperation. The European Union has generated initiatives to foster data openness and exchange, resulting in strategies like “ Science 2.0”, “ Open Research Data” and “ Open Science”.

If these initiatives become successful by networking different ecosystems for the benefit of their participants, they will generate an immediate competitive advantage because research and innovation processes are increasingly taking place in cooperative value chains. Thus, it is important to emphasise that value chains cannot be developed in isolation, but are rather a global endeavour, and their success depends on open access to sources of a wide variety of knowledge.

To address the opportunities and challenges derived by new technological developments, governments and funding agencies promote the development and use of collaborative ways to produce and share knowledge and data as early as possible in the research process, but also to appropriately secure results. The EU has established the European Open Science Cloud as a constituting element that is intended to provide “.... access and reliable reuse of research data to European researchers, innovators, companies and citizens through a trusted [infrastructure]”. This strategy aims to enable science to become more efficient through better sharing of resources, more reliable through better validation procedures, and more responsive to society’s needs.

To facilitate research & innovation (R&I), one crucial step is to ensure high quality of the research data. High quality can only be achieved if data is generated according to well-defined and validated methodologies, using standards and proper means of quality control (QC). Most importantly, the use of standards and QC procedures must be applied consistently by the whole community for the data to be reproducible, easily shared and reused.

The “reproducibility crisis” within the life science research is a problem that emerged in recent years ^1^. There are alarming reports from established representatives, like the Global Biological Standard Institute that in some fields such as cancer and preclinical research the majority of the published results is not reproducible ^2^. The reasons for this “reproducibility crisis” are manifold, among them the lack of appropriate study design, proper controls, or insufficient documentation, etc. But the main reason is the absence of a unifying quality control and assurance framework. The ignorance of this aspect comes at high costs for the society, as recently stated “ *A 50% irreproducibility rate within preclinical research implies that more $28B/year is spent on research that is irreproducible”* ^3^. Properly established research standards advance the alignment of consensus-based best practices, reduce variance, and improve reproducibility and quality in research.

A lack of interoperability is another factor that is currently often limiting the exchange and use of data from different sources. The need for data and tool interoperability and open standards to allow source connection was identified years ago. Still, only in recent years, the research community is pushing actors to develop proper tools and implement the use of standards. As summarised in an article of Sansone and Rocca-Serra in 2016, “...interoperability standards [should be recognised] as digital objects in their own right, with their associated research, development and educational activities” ^4^. Full implementation of interoperability has not yet been achieved but needs to be fostered to tap the full potential of existing resources.

It could all be easy, but it is not. Detailed metadata descriptions and detailed annotations are required to enable and accelerate the desired innovation process. In life science research, there is a variety of languages, vocabularies, formats and methods that must be used, and not all necessary standards or tools that facilitate their automatic conversion are currently available. New technologies introduced in the last decade brought the flourishing of many new omics disciplines and multidisciplinary research approaches. This inherent heterogeneity of data sources and competencies now requires specific actions for the development and uniform adoption of standard procedures to be followed for enhanced reproducibility, sharing and reuse ^5^. These actions are essential for advancing science and the utilization of research results for economic and societal benefits.

## The CHARME community

The network of the COST Action CHARME (CA15110) is composed of a team of experts from different sectors of life science research and standardisation across 32 countries in Europe and beyond, has been working during 2016–2020 aiming to increase awareness for the need for standards, and identify critical existing gaps that need to be closed.

After four years of successful work, the members of CHARME met in Brussels to summarise the achievements, to discuss future perspectives and challenges for standardisation in the life sciences and to elaborate a strategy to be suggested to the EC to accelerate the process of a wider application of standards in the life science research domain.

Following the motto “Standards make the world go round”, the outcomes of the COST Action are manifold and introduced some basic concepts and definitions that support a better understanding of the challenges and requirements. However, these endeavours need to be accompanied by adequate policies. In the next section, we provide a list of challenges we have identified in the frame of standardisation that are key to improve the quality and innovation potential of the European life science research.

### Communication

The first challenge identified is that the knowledge of standards and standardisation urgently need advertising and consequent awareness of the importance of their use. Many scientists are not aware of the standards in their field and suspect that the use of standards will limit their scientific freedom. It is exactly the opponents of standardisation that unconsciously use and appreciate these standards every day. Examples include operative systems like Android, formats like xml, pdf, jpg and QR- and barcodes, etc. It is essential for us to communicate the benefits of standards in the life sciences and the risks if standards are not acknowledged.

### Reproducibility and quality control

The reproducibility crisis as mentioned above ^3^ is another challenge that needs action. Reasons for this crisis are manifold, among them the lack of appropriate study design, proper controls or insufficient documentation, etc. But the main reason is the absence of a unifying quality control and assurance framework. High-quality records are essential for the quality and reproducibility of research results and efficient technology transfer. Standards facilitate the alignment of consensus-based best practices, reduce variance, and improve reproducibility in research.

Another important issue is the verifiable origin of data. It is a crucial point for researchers but also for biotechnology companies. The documentation acts as a certificate for potential users (customers) and improves the traceability and transparency of the research process intending to prove the reliability of results. A good starting point for making a change is the introduction of quality documentation of experiments which is frequently an obvious lack within the process.

Finally, to generate accurate and reproducible data sets for inter-laboratory comparisons, as well as further and future use of research data, it is mandatory to work in line with well-defined and validated methodologies, in compliance with standards and, where appropriate, according to good laboratory practices (GLP) and to data management plans (DMPs) that are produced at the planning stage of the research cycle ^6^. Data management and stewardship are both concepts enforced by funding agencies in very recent years. Currently there is an overall lack of experts, and only a few universities in Europe have professional profiles and personnel that can cover the needs of their laboratories and computational teams with this specific expertise.

### Supportive tools and actions

Along the pipeline of acquisition of data to (re)use we need to implement standards for description of samples and experiments, and standardised processes in the research workflows from the beginning.

In the context of data acquisition and storage, the potency for interoperability and transferability between different data formats and tools is likewise also limited. Whereas massive efforts have been made at the metadata level (data management plans, FAIR Data Principles, etc.), in context with the tools that can allow the easy and fast exchange of data amongst different data platforms or laboratories or between both, are still limited to a few research areas of life sciences.

An example is represented by electronic lab notebooks. They are relevant instruments to support the implementation of standards in the daily practice guiding the research process documentation. The connection of diverse ELN systems amongst each other, the study capture databases and the data repositories, and the standard development needed for that, were one of the main outcomes of CHARME. Nevertheless, this is what we see as one of the areas, if not a major area, where further adaptation of standards will help most to get real data to reuse.

### Education and training

A key challenge is to identify and fill evident gaps in education and training. Who knows how a standard is generated or what the difference between de facto and de jure standard is? It is of high importance to educate researchers that will become “next-generation” scientists being aware of quality management and standardisation in research. Besides, training and raising awareness is needed on all levels within the academic (and non-academic) system. Standards and quality management shake hands. A young researcher should get in contact with both issues as early as possible in the career, potentially already during his or her Bachelor studies. Learning about the basics like standard operating procedures (SOPs), quality control (QC) and data management should become as crucial as learning experimental design and how to document results.

Because the topic of implementation of standards is relatively new, and the awareness of its importance is rising in the scientific community, it is important to ensure proper training programmes for the university teachers. “Train-the-Trainer” programmes can multiply the effect to provide the necessary skills and knowledge to deliver courses on standards and quality management in research ^7^.

### Incentives and recognition

It is important to understand that standardisation does not necessarily mean the refinement or development of standards. Conversely and hopefully, whenever possible, standardisation rather means applying existing standards, whether de facto or de jure ^8^. The generation of standards in the sense of standardisation bodies like the International Organisation for Standardisation (ISO) or similar is a complex, time-consuming and cost-intensive process. In life science research and innovation processes, the development of an ISO standard often takes longer than the contract term of the project staff. Moreover, all the ISO standards should be reviewed every five years in terms of relevance to the marketplace.

To make matters worse, the use of standards typically requires the prior purchase of the document, making them very unattractive for most academic researchers. Another difficulty is the fact that there is currently no recognition for the contribution to the development of standards. Authors are not mentioned or acknowledged (i.e., the standards are created almost anonymously), and they do not count as a scientific track. Authorship recognition also affects the publication of raw data uploads to public repositories. Decision-makers, funding agencies and institutions urgently need to rethink if science should adopt and contribute to the overall process.

Incentives should also be available for those academic or research institutions that profuse efforts toward the creation of virtuous research value-chains to make their research innovative and of high quality. This implies to invest in personnel and technologies for the development of projects and the production and management of data in line with FAIR principles and Open Access. Here we stress the needs and recommendations in the document “Policy Recommendations - Cost-Benefit analysis for FAIR research data” ^9^. In this document, the section “Secure public funding for implementing and sustaining FAIR research data implementation” includes, among others, the need for FAIR-related costs, such as data stewardship and management, or data infrastructure operational costs to be made eligible for public funding. Specifically, Rec. 27 asks for: *“research funding for FAIR data should continue being available not only at the European level, for example as part of Horizon 2020 and Horizon Europe, but also as part of national funded research and innovation programmes”*.

On the overall, being the standards at the basis of FAIR data principle application, a specific focus on all the aspects related to the cost of their implementation also in terms of human resources should be taken under serious consideration.

### Investment vs impact

The insufficient quality of the published data in the life-sciences tremendously reduces public investment in research. Current evaluation of the published data is leading to a worst-case scenario, e.g. for the medical sciences that up to 80% of the available data are not reusable. To avoid such waste, standards need to be developed and implemented for the entire research life cycle. Achieving a long-term and sustainable improvement requires not only awareness, engagement, training and education, but also an investment in infrastructure and personnel. Technical infrastructure needs to be established, maintained and constantly updated to allow an efficient and secure storing/hosting of the data generated by the scientific community. Public repositories have already been established offering the archiving of scientific data for all disciplines like OpenAIRE or specific research fields, like SEEK for the medical sciences. These repositories are only valuable if the data and associated metadata are of sufficient quality and following minimal standards. Therefore, also internal technical infrastructure hosted by research institutes should be established in a way that standards are applied. For providing technical solutions, but also for training, on-site advice and help, additional investments in personnel are required.

The sharing of medical (patient) data is an issue that is highly relevant in the context of Open Science and data sharing but needs to be discussed more intensively elsewhere. Nevertheless, it should be noted here that the strict ethical laws and regulations limit the sharing of patient-derived data – even if anonymised and collated. The availability of these type of resources are a wealth of information that – if made available – could accelerate the research process and avoid duplication of efforts. New paradigms that enforce a participatory approach and active involvement of patients might be a route to solve this problem. Standards are a non-negotiable prerequisite for such an innovative approach.

Considering that a broader implementation of standards within the scientific system might need long-term investments and commitment to introduce a change in how we perform science, it must also be considered what we put at risk:

*“Interpreting the overall cost of not having FAIR research data as a single value overlooks many non-quantifiable benefits of FAIR. Nonetheless, at €10.2bn per year in Europe, the measurable cost of not having FAIR research data makes an overwhelming case, in favour of the implementation of the FAIR principles*” ^9^.

Although most stakeholders would agree that the way we perform research must change, the questions of who has the responsibility (and power) to introduce the change within the system and how we can finance an efficient support system are open to debate.

There is a need for European decision-makers to implement actions supporting all areas of the life science research (academic and non-academic) for equal opportunities and access to technology infrastructures, education programmes, and funding.

## CHARME outcomes and discussion

*“Making a step towards a European concept for excellence and trust in life science research”.*

Improving access to and management of data is fundamental, and we see that the importance of standardisation approaches for data collection, data description, application of the FAIR principles and for data modelling is increasingly recognised. But many of these efforts are still scattered, the awareness for available solutions is scarce, and there is missing recognition that standards still need to be harmonised, connected and further developed to make the system efficient. In general, this is about harmonising the field to maximise the way we benefit from already ongoing activities. That still needs better integration of ongoing initiatives on all the aspects mentioned.

In response to these challenges and to ensure the high quality of life science research data and their usability for R&I, measures must be taken at several levels to build an ecosystem of excellence and trust. Here we describe a series of measures that the CHARME network has identified as essential to this aim.

**Measure 1: Securing access to data and computing infrastructures with harmonised solutions for standardisation processes across disciplines and countries**

Connection of existing and new local, national, European and international services into one interacting end-user service system, which should also strive for harmonisation with non-European efforts. That is, developing a structure in which communities jointly define standards according to standardisation bodies and guidelines from existing standardisation initiatives. This system should include the collection of information on standards and SOPs from different domains and enabling interaction with existing frameworks for agreement on nomenclature, ontologies and their international adoption. Integration and connection of existing expertise and services avoid heterogeneity of activities and ensures inclusiveness of all actors. Directly related to the need for connection and interaction between the existing facilities is the agreement about standard formats and operational procedures to enable reuse and reproducibility of data.

Experts on this service structure should formulate advice to roadmapping efforts to indicate where standards need to be linked, identify overlaps and what kind of interoperability services still need to be developed. This structure should include a helpdesk and advice centre providing support and information advice that can point people to the relevant aspects but also a collection of standards and SOPs for different research domains. Several examples of suitable solutions for the use of SOPs in different life science domains already exist. For example, huge efforts have been made in the systems biology field for modelling and data exchange such as COPASI, CellNetAnalyzer, SABIO-RK and the SEEK platform of FAIRDOMHUB. The SEEK platform is a web-based resource for sharing diverse scientific research datasets, models or simulations, processes, protocols, SOPs and research outcomes. Another important initiative is EOSC life which is expected to act as an umbrella project for initiatives and projects like ELIXIR-Converge, the various life sciences related ESFRI and IMI projects and the GO-FAIR implementation network.

**Measure 2: Establishment and support of training networks and infrastructures to enable capacity building on standards, SOPs, and data management across all career levels, institutions, and countries**

There are already many ongoing training initiatives (e.g., TeSS, the ELIXIR’s Training Portal, Train at EMBL-EBI, The Carpentry, GOBLET, FAIRsFAIR, etc.) and mostly every larger research project has its training work package or training module. Each of them provides training for their specific community. Although these training activities are highly relevant with an increased reach, they are not sufficient to address the entire research community and cover the specificities of the broad spectrum of the life science fields. To solve this, we need to join efforts and develop a common capacity building framework by coordinating training activities led by international, high quality, collaborative Science & Technology (S&T) networks. This will bridge and connect research disciplines also content-wise and link the standardisation approaches they use. This will also enable interdisciplinary cooperation by the integration of expertise from nearby research sectors/communities in life-science research (systems biology, systems medicine, medicine, biotechnology, plant science, computational biology and bioinformatics, etc.), and allow breakthroughs in scientific development built on synergistic efforts.

More specifically, the following measures must be addressed:

• Concerted training actions facilitating the **expansion of the currently sparse critical mass of specialists and experts** who understand and possess knowledge of the principles and relevant tools within Europe. Cooperation of world-leading experts within each scientific field thereby will ensure mutual benefits and the best possible platform for any research and educational activities. Current collaborations among European and non-European organisations are e.g., the CABANA project, GOBLET-EMBnet and ISCB (North America), SoIBio (in South America), ApBioNet (Asia), ASBCB and H3ABioNet (Africa).

• **Train-the-Trainer**. A major effort should be made for enlarging the offer of train-the-trainer programmes to allows trainers to acquire good training practices embracing standards ^7^. Such action would enforce and multiply the spreading of knowledge in good practices for life science research and computational biology in the academic world and beyond.

• **Curricula**. Besides offering training modules in undergraduate and/or graduate programmes, additional approaches might be worthwhile to consider engaging young scientists in the appreciation and use of standards. Students might be offered the possibility to participate in standards-related activities, e.g. in programming sessions. For example, COMBINE (“COmputational Modeling in BIology Network)” is an initiative to coordinate the development of the various community standards and formats for computational models. Alternatively, they could be involved in the development and testing of SOPs. Such activities should be linked to a credit point system incentivising their contribution. Benefits might be manifold: the students have the chance to learn hands-on with experts, they might even have the chance to be an author in a publication, the university would gain new knowledge and expertise, and the standardisation community is sustainably supported by engaged young researchers.

• **New professorships.** Establishment of a research agenda in the field of standardisation and innovation transfer by (endowed) chairs on standardisation and innovation throughout Europe.

• **Train the experts.** Training in standards, standardised processes and standardisation frameworks should also include training of established researchers and supervisors because they are often acting as reviewers and evaluators and should be sensitised for the topic and appreciate the relevance.

Any construct that provides activities as mentioned above in research, education, storing services and that is willing to interact and become a recognised member of the community should be exclusively designated following the measure pointed out in Measure 3.

**Measure 3: Roadmap for the implementation of formal quality management systems (QMS)**

We think that there is a strong need for mechanisms of control for the quality of openly accessible data. This data check must be upstream of the open access. A “ *seal of quality*” similar to a DMP with a clear definition of quality benchmarks for data is needed in order to define metrics which are applicable and reasonable for building a framework around good data quality ^10^ which are unthinkingly usable for further proceeding by everyone. This *seal of quality* should be supported by incentives from funders and publishers. Incentives should also consider another important aspect, that is education to acceptance of quality assurance (QA) plans. There are many examples of resistance of researchers to accept rules that QA plans impose and how these resistances can be easily overcome by education ^11^. As indicated above, course programs and educational material on topics like standard operating procedures (SOPs), quality control (QC) and data management need to be developed and implemented as part of university curricula and career development modules. Together with “Train the Trainer” measures, these educational programs will be a crucial step towards a safe and reliable way to do research.

• **Introduction of measures for QMS in funding programs.** QMS will ensure quality assurance and reproducibility of research data which are prerequisites for all knowledge transfer activities in life-science research, and thus will have a substantial impact on technology transfer. This will contribute to increase and foster interdisciplinary and transnational collaboration between stakeholders from academia and industry and create a network with strong potential to impact and address social, ecological, economic challenges. The introduction of QMS should be done in a top-down approach like the implementation of DMPs, at a first stage voluntarily and connected to incentives and recognition for scientists who follow the principles *.*

• **Awarding a “Seal of quality”** by the development and application of a review mechanism system that selects, appraises and monitors the performance of institutions, centres, platforms, or infrastructures. Such an accreditation-like system would ensure that high-quality performance of institutions in their implementation of internal policy and provision of infrastructure for data management and standards are visible and acknowledged. 

A best practice model for such a system is the Organisation of the European Cancer Research Institutes (OECI), who developed a catalogue of criteria that ensures high quality research from the participating institutions. Now all cancer research institutes and university clinics need to have at least a minimum of criteria to be fulfilled to be part of the OECI. This seal of quality could not only become an indicator for the researcher’s selection and decision for future employers but may also become a criterion for faster acceptance of publications. The same rating approach should be applied to datasets. Datasets frequently used by the research community and found to be reliable in terms of their FAIRness and reproducibility should get high ratings. Researchers should be able to share their experience when they use a particular dataset. This system would also be transferable to computational models to ensure reproducibility of the modelling based on the model parameters given and the dataset provided. For example, the MAQC society is now focused on establishing evaluation systems for the reproducibility of computer models based on first transcriptomics data. In future, they will extend these efforts to other omics data as well.

**Measure 4: Fostering interaction with the non-academic sector for the cross-fertilisation of ideas, for shaping knowledge and expertise, to reduce costs, and to streamline technology transfer**

Standards are important aspects in industry and ISO was founded with the idea of answering a fundamental question: “ what’s the best way of doing this?”. Standards in life sciences are not only supporting knowledge and data exchange, but they are also ensuring the reliability of the materials, products, methods, or services and thus help to ensure product functionality, and support consumer safety and public health.

Co-creation processes in standardisation are of mutual benefit and support knowledge and technology transfer, open innovation and cross-industry innovation. Although many standards have been developed in recent years, the insufficient implementation of and compliance to existing standards, e.g. within the life science sector, lead to a disruption of the innovation pipeline - often happening at the interface between academic research and industry - simply because of bad quality and missing reproducibility/reusability of the data.

Academics are often not aware of the necessity of standardised processes, especially in application-oriented work, which negatively affects or prevents academia-industry cooperation. In the development of drugs or new diagnostic tools, this ignorance to apply standards and quality management strategies leads to unnecessary disruption in the process because many results cannot be reproduced.

Thus, to facilitate the open innovation processes and to improve the collaboration between groups in academia and industry, partners from all sectors and stakeholder groups need to be involved as much as possible in standardisation activities launched by standardisation bodies. Still, also their voice needs to be considered in academia-driven grassroot standardisation initiatives.

Education in the field of standardisation at universities in the life sciences, management sciences, economics and law, therefore, is a key strategy to enable both, successful academia-industry cooperation and generation of innovation. A good example here is the Master’s course “ Technopreneurship” (MTECH) at the University of Luxembourg aiming at the education of students to transfer smart secure ICT knowledge directly into technical innovation, through the prism of the competitive and innovative tool of technical standardisation.

Finally, appropriate education in the context of standardisation leads to another advantage: skills in standardisation processes increase job opportunities in the non-academic sector. As mentioned already, knowledge about regulations and standards is crucial components to ensure best practice and help to ensure product quality and consumer safety. Therefore, it seems beneficial to:

- involve non-academic partners in the development of curricula at the undergraduate and graduate levels based on market needs;
- implement mandatory seminars, colloquia provided by non-academic partners as well as study visits and traineeships in the industry into the curricula and the examination infrastructure;
- establish standardisation and innovation transfer chairs.

**Our vision: “ *A global umbrella infrastructure-helpdesk on standardisation”***

Based on the experience gained within the COST Action CHARME, we suggest the establishment of an umbrella hub (helpdesk) on standardisation. Similar to the IP Helpdesk of the European Commission, this construct will bundle and harmonise all initiatives and institutions that are already focussing on this topic and will represent a central hub for all activities related to standards and standardisation.

This hub should:

1. Consist of experts from the different fields, institutions, bodies and initiatives and bodies in life science research mentioned above, and offer services to answer all the questions related to SOPs, protocols and data standards.

2. Provide Open Access protocols, methods and tools serving as a basis for proper data management (SOPs, Standards) by establishing an Application Program Interface (API) to a queryable and searchable database of existing standards, SOPs, protocols and tools with links to the corresponding guardians, and actors and mechanisms for collaborative preparation.

3. Adopt EU- wide training by providing Train-the-Trainer modules and sharing of high-quality teaching materials and methods among education professionals across borders.

4. Include budget for funding programmes or projects, e.g. for the generation of tools enabling (cross-sectoral) interoperability and training.

5. Become the contact point for the communication and collection of needs from the operational level, but also for translation and exchange with decision-makers.

6. Be open to the public and free of charges, centralised and monitored by the EC.

7. Become the core of a worldwide infrastructure and thus function as an integrators between grassroot scientific initiatives and activities governed by standardisation bodies.

## Conclusions

We believe that this White Paper demonstrates the global need to promote standards in the life sciences research in response to a major challenge of implementing open science principles in the academic workflow, especially with respect to the reproducibility and reliability of research data. To promote these standards, we have identified four measures to support their implementation across the entire research community. Interactions with platforms and communities such as ELIXIR and worldwide integration in the complex landscape of societies, initiatives and projects have been addressed to avoid duplication of efforts and ensure fruitful collaborations. Finally, the growing interest for reproducible data will ensure the global recognition and expansion of the research community and will trigger numerous novel interactions between academia and industry. Europe is well-positioned for global leadership in building alliances around common values, and promoting FAIR principles and standardisation processes and developing a landscape of interoperability services to facilitate these connections.

## Data availability

No data is associated with this article.

## Disclaimer

W.T.: The views presented in this article do not necessarily reflect current or future opinion or policy of the US Food and Drug Administration. Any mention of commercial products is for clarification and not intended as an endorsement.
