# Supplementary material for: The need for standardisation in life science research - an approach to excellence and trust
Source: F1000Res. 2021 May 10;9:1398. Originally published 2020 Dec 4. [Version 2] doi: 10.12688/f1000research.27500.2 (PMC7863991; doi:10.12688/f1000research.27500.2)
Supplement: Peer review history

# Peer review: Reviewer response for version 2

**DOI:** 10.5256/f1000research.56312.r85009

**Published:** 2021-05-12

**Authors:** David Nickerson

**Affiliations:** 1 Auckland Bioengineering Institute, University of Auckland, Auckland, New Zealand

No further comments.

Is the topic of the opinion article discussed accurately in the context of the current literature?

Yes

Are arguments sufficiently supported by evidence from the published literature?

Yes

Are all factual statements correct and adequately supported by citations?

Yes

Are the conclusions drawn balanced and justified on the basis of the presented arguments?

Yes

Reviewer Expertise:

Computational physiology; reproducibility; modelling and simulation standards; model repositories; semantic annotation.

I confirm that I have read this submission and believe that I have an appropriate level of expertise to confirm that it is of an acceptable scientific standard.

# Peer review: Reviewer response for version 1

**DOI:** 10.5256/f1000research.30393.r77735

**Published:** 2021-02-08

**Authors:** Antonella Lanati

**Affiliations:** 1 Valore Qualità, Pavia, Italy; 2 Vita-Salute San Raffaele University, Milan, Italy

In the opinion paper “The need for standardisation in life science research - an approach to excellence and trust”, the authors suggest and discuss measures to ensure high quality and reusability of data in life science research.

 A quality approach, to facilitate research and innovation and to respond to “reproducibility crisis”, is deployed in various aspects and at different levels, from dedicated training and need for Quality Control, up to compliance with international standards and requirements for effective communication. Attention is also paid to the problem of coordination and standardization in research, addressed by quality generation and processing of data, and by adoption of commonly recognized protocols of communications. Particular emphasis is given to the need for recognition and support to free international standards, as the foundation to implement open science principles in research.

 The paper is very in-depth discussed, wide-ranging and complete, with sharp observations and concrete proposals. Due attention is given not only to the main standardisation needs, but also to specific aspects regarding the stewardship and the management of scientific research.

 This paper covers the increasing interest for reproducible data and can thus provide a useful contribution to spreading knowledge and principles of research reliability in the scientific community.

 I have no major concerns about the paper, I would suggest only few minor changes:

1. Avoid the repetition of few sentences regarding the reproducibility crisis: the topic in page 3, II column, from 14th line on, could be just outlined there and further discussed with the support of citations in page 4, II column, from line 7 on.
2. The chapter “education” might benefit from moving after “Reproducibility and quality control” and “Supportive tools and actions”, i. e. after having introduced the problem of the lack of management culture and the need for figures with specific preparation.
3. The Measure #3 may include also training on soft skills, as suggested by the needs for education previously highlighted.

Is the topic of the opinion article discussed accurately in the context of the current literature?

Yes

Are arguments sufficiently supported by evidence from the published literature?

Yes

Are all factual statements correct and adequately supported by citations?

Yes

Are the conclusions drawn balanced and justified on the basis of the presented arguments?

Yes

Reviewer Expertise:

Quality management in scientific research

I confirm that I have read this submission and believe that I have an appropriate level of expertise to confirm that it is of an acceptable scientific standard.

# Response

**Published:** 2021-04-20

**Authors:** Susanne Hollmann

**Affiliations:** University of Potsdam, Germany

The authors thank you very much for reviewing and commenting our manuscript. We prepared a revised version of the manuscript based on your and the feedback of the other reviewers.

**Avoid the repetition of few sentences regarding the reproducibility crisis: the topic in page 3, II column, from 14th line on, could be just outlined there and further discussed with the support of citations in page 4, II column, from line 7 on.**

 Thank you for spotting the repetition in the two instances. We changed the text following your recommendation to:

 “Reproducibility and quality control

 The reproducibility crisis as mentioned above ^2^ is another challenge that needs action. Reasons for this reproducibility crisis are manifold, among them the lack of appropriate study design, proper controls or insufficient documentation, etc. But the main reason is the absence of a unifying quality control and assurance framework. High-quality records are essential for the quality and reproducibility of research results and efficient technology transfer. Standards facilitate the alignment of consensus-based best practices, reduce variance, and improve reproducibility in research.

**The chapter “education” might benefit from moving after “Reproducibility and quality control” and “Supportive tools and actions”, i. e. after having introduced the problem of the lack of management culture and the need for figures with specific preparation.**

 We appreciate the recommendation and moved the chapter “Education and training” and included it after the section on “Supportive tools and actions”.

 In the chapter “Education and training” we also changed the wording of the first sentence to:

 “A key challenge is to identify and fill evident gaps in education and training.”

**The Measure #3 may include also training on soft skills, as suggested by the needs for education previously highlighted.**

 This is an excellent recommendation. We changed the text for measure 3 as follows:

 “…There are many examples of resistance of researchers to accept rules that QA plans impose and how these resistances can be easily overcome by education ^11^. As indicated above, course programs and educational material on topics like standard operating procedures (SOPs), quality control (QC) and data management need to be developed and implement as part of university curricula and career development modules. Together with “Train-the-Trainer” measures, these educational programs represent a crucial step towards a safe and reliable way to do research.”

# Peer review: Reviewer response for version 1

**DOI:** 10.5256/f1000research.30393.r77734

**Published:** 2021-02-04

**Authors:** Laurent Falquet

**Affiliations:** 1 SIB Swiss Institute of Bioinformatics, Lausanne, Switzerland; 2 University of Fribourg, Fribourg, Switzerland

The authors clearly describe the current status of data and tools standards in the Life Sciences. They propose diverse actions to improve and promote the acceptance and usage of standards. 

 The manuscript points correctly to the metadata issues for which a big effort must be made to improve the meaningful reuse of the data.

 There could have been more emphasis on specific issues of medical data where the ethic agreements signed by the patients and the researchers often prevent the open distribution of the data and associated metadata even when anonymized. This a recurring concern affecting the reproducibility of the results.

Is the topic of the opinion article discussed accurately in the context of the current literature?

Yes

Are arguments sufficiently supported by evidence from the published literature?

Yes

Are all factual statements correct and adequately supported by citations?

Yes

Are the conclusions drawn balanced and justified on the basis of the presented arguments?

Yes

Reviewer Expertise:

Bioinformatics, microbial genomics and metagenomics.

I confirm that I have read this submission and believe that I have an appropriate level of expertise to confirm that it is of an acceptable scientific standard.

# Response

**Published:** 2021-04-20

**Authors:** Susanne Hollmann

**Affiliations:** University of Potsdam, Germany

Thank you very much for reviewing and commenting our manuscript and for drawing our attention to this essential aspect. We prepared a revised version of the manuscript based on the feedback of all reviewers.

**There could have been more emphasis on specific issues of medical data where the ethic agreements signed by the patients and the researchers often prevent the open distribution of the data and associated metadata even when anonymized. This a recurring concern affecting the reproducibility of the results.**

 Under the chapter “Investment and impact” we added the following new paragraph:

 “The sharing of medical (patient) data is an issue that is highly relevant in the context of Open Science and data sharing but needs to be discussed more intensively elsewhere. Nevertheless, it should be noted here that the strict ethical laws and regulations limit the sharing of patient-derived data – even if anonymised and collated. The availability of these type of resources are a wealth of information that – if made available – could accelerate the research process and avoid duplication of efforts. New paradigms that enforce a participatory approach and active involvement of patients might be a route to solve this problem. Standards are a non-negotiable prerequisite for such an innovative approach.”

# Peer review: Reviewer response for version 1

**DOI:** 10.5256/f1000research.30393.r75752

**Published:** 2021-02-01

**Authors:** David Nickerson

**Affiliations:** 1 Auckland Bioengineering Institute, University of Auckland, Auckland, New Zealand

In this opinion article the authors outline many significant scientific advances which are primarily driven by, and possible due to, advances in digital technologies (ICT). They then explore how full realisation of the benefits from these developments depends on open and transparent sharing of knowledge, data, models, etc.

 The authors approach this discussion from a European Union perspective, in the context of EU policies and projects, but touch of a range of non-EU initiatives. Indeed, the issues addressed are globally relevant and important.

 In particular, the authors summarise the outcomes from the COST Action CHARME by way of framing the prevalent challenges to adoption of suitable standards in life science research. This is followed by the measures the CHARME network has identified in response to these challenges and to ensure high quality life science research data that facilitates research and innovation.

 The authors conclude with a vision for a central EU umbrella hub (helpdesk) on standardisation to implement the discussed measures. Such a hub could lead the way with the EU providing global leadership in the adoption of the FAIR principles and standardisation processes and associated services and policies.

 This reviewer strongly supports the presented measures and the author's goals for improving the reproducibility and FAIRness of the life sciences.

 I only have a few very minor corrections to suggest just to tidy up the article.

Abstract:

- *FAIR Principle **s**.*

Introduction:

- Consistent use of "e.g.," or "e.g.".
- Perhaps a missing citation indicated by the "[citation in the note]" text?
- *"The ignorance of this aspect **coms** at high for the society, as recently"* - spelling and grammar issues in this sentence.
- *"Properly established research standards the alignment of consensus-based best practices, reduce variance, and improve reproducibility and quality in research."* - this sentence doesn't make sense to me, but not quite sure how to fix it. 

Is the topic of the opinion article discussed accurately in the context of the current literature?

Yes

Are arguments sufficiently supported by evidence from the published literature?

Yes

Are all factual statements correct and adequately supported by citations?

Yes

Are the conclusions drawn balanced and justified on the basis of the presented arguments?

Yes

Reviewer Expertise:

Computational physiology; reproducibility; modelling and simulation standards; model repositories; semantic annotation.

I confirm that I have read this submission and believe that I have an appropriate level of expertise to confirm that it is of an acceptable scientific standard.

# Response

**Published:** 2021-04-20

**Authors:** Susanne Hollmann

**Affiliations:** University of Potsdam, Germany

The authors thank you very much for reviewing and commenting our manuscript.

 Based on your and the feedback of the other reviewers we prepared a revised version of the manuscript.

**Consistent use of "e.g.," or "e.g.”**

 We use now consistently “e.g.” without commats as it refers to the British English style.

**Perhaps a missing citation indicated by the "[citation in the note]" text?**

 The indication for the reference was eliminated. The corresponding reference is given in the consecutive sentence and refers to reference 1:

*Freedman LP, Inglese J: The increasing urgency for standards in basic biologic research.* *Cancer Res.* *2014; **74**(15): 4024–9.*

***"The ignorance of this aspect coms at high for the society, as recently"* - spelling and grammar issues in this sentence.**

 The sentence has been changed to:

 “The ignorance of this aspect comes at high costs for the society, as recently stated…”

***"Properly established research standards the alignment of consensus-based best practices, reduce variance, and improve reproducibility and quality in research."* - this sentence doesn't make sense to me, but not quite sure how to fix it.**

 The sentence was changed adding “advance” to complete the sentence:

 “Properly established research standards *advance* the alignment of consensus-based best practices, reduce variance, and improve reproducibility and quality in research.”
